# Supplemental *Bacillus subtilis* DSM 29784 and enzymes, alone or in combination, as alternatives for antibiotics to improve growth performance, digestive enzyme activity, anti-oxidative status, immune response and the intestinal barrier of broiler chickens

**DOI:** 10.1017/S0007114520002755

**Published:** 2021-03-14

**Authors:** Yuanyuan Wang, Chianning Heng, Xihong Zhou, Guangtian Cao, Lei Jiang, Jiangshui Wang, Kaixuan Li, Dianchun Wang, Xiuan Zhan

**Affiliations:** 1 College of Animal Sciences, Zhejiang University, Hangzhou, People’s Republic of China; 2 Institute of Subtropical Agriculture, Chinese Academy of Sciences, People’s Republic of China; 3 College of Standardisation, China Jiliang University, Hangzhou, People’s Republic of China

**Keywords:** Lingnan yellow broilers, Antibiotics, *Bacillus subtilis* DSM29784, Intestinal barrier, Anti-oxidative capacity, Immunity capacity

## Abstract

The present study investigated the effect of *Bacillus subtilis* DSM 29784 (Ba) and enzymes (xylanase and *β*-glucanases; Enz), alone or in combination (BE) as antibiotic replacements, on the growth performance, digestive enzyme activity, immune response and the intestinal barrier of broiler chickens. In total, 1200 1-d-old broilers were randomly assigned to five dietary treatments, each with six replicate pens of forty birds for 63 d as follows: (a) basal diet (control), supplemented with (b) 1 × 10^9^ colony-forming units (cfu)/kg Ba, (c) 300 mg/kg Enz, (d) 1 × 10^9^ cfu/kg Ba and 300 mg/kg Enz and (e) 250 mg/kg enramycin (ER). Ba, Enz and BE, similar to ER, decreased the feed conversion rate, maintained intestinal integrity with a higher villus height:crypt depth ratio and increased the numbers of goblet cells. The BE group exhibited higher expression of claudin-1 and mucin 2 than the other four groups. BE supplementation significantly increased the *α*-diversity and *β*-diversity of the intestinal microbiota and markedly enhanced lipase activity in the duodenal mucosa. Serum endotoxin was significantly decreased in the BE group. Compared with those in the control group, increased superoxide dismutase and glutathione peroxidase activities were observed in the jejunal mucosa of the Ba and BE groups, respectively. In conclusion, the results suggested that dietary treatment with Ba, Enz or BE has beneficial effects on growth performance and anti-oxidative capacity, and BE had better effects than Ba or Enz alone on digestive enzyme activity and the intestinal microbiota. Ba or Enz could be used as an alternative to antibiotics for broiler chickens.

For more than 60 years, sub-therapeutic levels of dietary antibiotics have been used in the livestock and poultry industry to improve growth performance and prevent diseases^(1,2)^. Antibiotics stabilise the bacterial population present in the digestive tract resulting in improved performance and reduced morbidity and mortality due to clinical and subclinical diseases^(3)^. Enramycin (ER) is a large molecule polypeptide antibiotic effective against pathogens of the gut flora^(4)^ and exhibits a strong inhibitory effect against *Clostridium perfringens* ^(5)^, the pathogen responsible for inducing necrotic enteritis in chickens^(6)^.

Although this has been beneficial for animal health and productivity, it has essentially been a double-edged sword as antibiotic resistance and antibiotic residues in animal products threaten human health^(7)^. In response to this situation, antibiotic usage as growth promoters in feed has been banned in the European Union and there is increasing pressure for stricter regulations in North America^(8)^. A ban on feed antibiotics will be implemented in China in 2020. However, limitation of antibiotics has led to problems such as reduction in animal performance and feed conversion, and an increased incidence of certain animal diseases^(9,10)^. Therefore, exploring alternatives to antibiotics is an urgent task. Researchers are focusing on alternatives to antibiotics to improve animal performance and optimise gut health^(11)^. These alternatives include acidifiers (organic acids), prebiotics, probiotics, enzymes, herbal products, microflora enhancers and immune modulators.

The use of probiotics in broiler diets is a strategy that can improve diversity and stability of the intestinal microbiota, immunomodulation, competition for adhesion sites and the production of antimicrobial agents resulting in improved broiler performance^(12–14)^. In addition, accumulating studies indicate that probiotics exert beneficial effects on body anti-oxidative capacity, digestive enzymes and nutrient absorption and decrease cell apoptosis, which finally favours the gut health and production performance of broilers^(15–18)^. Among many bacterial species used as probiotics, spore-forming *Bacillus* spp. has been identified as a suitable probiotic owing to the resistance of its spores to harsh conditions and long-term storage at ambient temperature^(19,20)^. *Bacillus subtilis* is not a normal intestinal microorganism but rather is a facultative anaerobe that can grow in the gut^(21)^. *Bacillus subtilis* DSM 29784 (Ba) was originally isolated from soil and deposited in the Deutsche Sammlung von Mikroorganismen und Zellkulturen. Previous studies have demonstrated that Ba can improve growth performance and gut health in pullets^(22)^ and turkeys^(23)^. However, there is little experimental research on the effect of Ba on the antioxidant status, immune response and gut microbiota of broilers.

Currently, non-conventional feed ingredients, such as barley and by-products from biofuel industries and other agro-industries, are being used as an alternative feedstuff to reduce the cost of broiler feed production^(24)^. However, these feed ingredients are inherently high in non-starch polysaccharides. These non-starch polysaccharides produce an anti-nutritive effect by increasing viscosity and entrapping nutrients in digesta^(25)^. Broilers lack endogenous enzymes required for non-starch polysaccharides digestion and thus exhibit reduced feed efficiency when fibre content is increased even in a nutritionally complete diet^(26)^. Supplemental xylanase and *β*-glucanase can disrupt the plant cell wall matrix by hydrolysing inaccessible carbohydrates^(27)^. Previous studies have documented that the use of xylanase and *β*-glucanase can partially hydrolyse non-starch polysaccharides, reduce the viscosity of gut contents and result in improved nutrient absorption^(28)^. Further, these enzymes could improve the growth performance and gut health, increase the villus size and villus height:crypt depth ratio and influence caecal microbiota^(29,30)^.

To the best of our knowledge, the combined use of exogenous digestive enzymes and probiotics as supplements in broilers has rarely been reported. However, combined supplementation of exogenous enzymes and probiotics could result in complementary modes of action. According to Adeoye *et al*.^(31)^, supplementation of the diet with a combination of enzymes and probiotics can improve gut health without deleterious effects on the intestinal microbial composition as probiotics have the ability to produce fibre-degrading enzymes that might complement endogenous enzyme activity. In addition, exogenous digestive enzymes could increase the availability of suitable substrates for the probiotic and improve the growth of other beneficial bacteria. Given the potential complementary modes of action of the exogenous digestive enzymes and probiotics, the two products could improve the growth performance and health status of broilers when fed diets supplemented with both enzymes (Enz treatment) and probiotics as a cocktail. Therefore, it was hypothesised that the combination of Ba and Enz (BE) might have a synergistic effect that could result in improved intestinal health and overall broiler performance. The present study aimed to assess the effectiveness of the Ba and Enz blend in comparison with that of ER on the growth performance, digestive enzyme activity, anti-oxidative status, immune response and the intestinal barrier of broiler chickens.

## Materials and methods

### Probiotics and enzyme preparation

The *B. subtilis* strain was originally isolated from soil and has not been GM. The strain has been deposited in the Deutsche Sammlung von Mikroorganismen und Zellkulturen with the accession no. DSM 29784. Ba was cultured on Luria–Bertani media, kept at 37°C for 24 h and shaken at 180 rpm. Pure bacterial cells were collected after centrifugation at 5000 ***g*** for 10 min at 4°C. Then, these cells were washed twice with sterile 0·85 % sodium chloride solution. Ultimately, the culture purity and identification were constantly checked via the spread plate method^(32)^. The exogenous enzyme complex primarily contained 3200 U/g *β*-glucanase and 6225 U/g xylanase. Xylanase and *β*-glucanase activities were determined as described by Cosson *et al*.^(33)^. The unit of activity was defined as the amount of enzyme that releases one micromole of reducing sugars per minute at a pH of 4–8 and a temperature of 50°C.

### Chickens, diet and management

A total of 1200 1-d-old Lingnan yellow broilers were randomly allotted to a total of thirty floor pens (area of 2 m × 4 m) covered with fresh wood shavings, and the total weight of forty chicks (twenty males and twenty females) per pen was equivalent. The birds in five treatment groups each consisted of six replicates with forty chicks per replicate. The birds were allowed *ad libitum* access to water and diets throughout (days 1–63) and were kept under light–dark (2L–1D) cycles every day. The control group was fed the basal diet and the treatment groups received (a) the same basal diet supplemented with (b) 1 × 10^9^ colony-forming units (cfu)/kg Ba powder, (c) 300 mg/kg Enz (3200 U/g *β*-glucanase and 6225 U/g xylanase), (d) 1 × 10^9^ cfu/kg Ba powder and 300 mg/kg Enz and (e) 250 mg/kg ER, for 63 d. The experimental diet was designed according to National Research Council requirements^(34)^. The composition and nutrient levels of the basal diets are shown in Table 1.

**Table 1.** Composition and nutrient level of the basal diet (% as fed basis)

Ingredients	Starter (1–21 d)	Grower (22–42 d)	Finisher (43–63 d)
Maize	58·00	40·00	42·00
Sorghum		16·00	17·00
Barley		10·00	13·00
Broken rice	5·00		
Soyabean meal	30·50	18·00	11·00
Maize gluten meal	2·00	5·00	5·00
Distillers dried grains with solubles		5·00	5·00
Lard		2·00	3·00
Soyabean oil	0·50		
Salt	0·14	0·11	0·10
Calcium hydrogen phosphate	1·20	1·00	0·80
Limestone	1·50	1·20	1·20
Zeolite		0·50	0·70
Premix*	1·00	1·00	1·00
Total	100·00	100·00	100·00
ME (MJ/kg)†	12·26	12·59	12·97
Crude protein	21·02	19·06	18·20
Lysine	1·09	0·98	0·86
Methionine	0·50	0·41	0·38
Methionine + cysteine	0·87	0·73	0·66
Ca	1·04	0·92	0·87
Total P	0·69	0·66	0·61

ME, metabolisable energy; V_A_, vitamin A; V_D3_, vitamin D_3_; V_E_, vitamin E; V_K_, vitamin K; V_B12_, vitamin B_12_.

* Supplied per kg of diet: from 1 to 21 d, Fe 80 mg, Cu 7 mg, Zn 85 mg, Mn 60 mg, iodine 0·35 mg, Se 0·23 mg, V_A_ 1·65 mg, V_D3_ 0·18 mg, V_E_ 20 mg, V_k_ 0·50 mg, V_B12_ 10 mg, thiamin 3·8 mg, riboflavin 4·0 mg, niacin 25 mg, pantothenic acid 10 mg, pyridoxine 3·5 mg, biotin 0·15 mg, folic acid 0·55 mg and choline 1·0 g; from 22 to 42 d, Fe 80 mg, Cu 7 mg, Zn 60 mg, Mn 60 mg, iodine 0·35 mg, Se 0·15 mg, V_A_ 1·05 mg, V_D3_ 0·12 mg, V_E_ 10 mg, V_k_ 0·50 mg, V_B12_ 10 mg, thiamin 2·4 mg, riboflavin 4·0 mg, niacin 17 mg, pantothenic acid 10 mg, pyridoxine 3·5 mg, biotin 0·15 mg, folic acid 0·55 mg and choline 0·75 g; from 43 to 63 d, Fe 80 mg, Cu 7 mg, Zn 60 mg, Mn 60 mg, iodine 0·35 mg, Se 0·15 mg, V_A_ 0·525 mg, V_D3_ 0·06 mg, V_E_ 10 mg, V_k_ 0·50 mg, V_B12_ 10 mg, thiamin 2·1 mg, riboflavin 3·0 mg, niacin 15 mg, pantothenic acid 10 mg, pyridoxine 3·5 mg, biotin 0·15 mg, folic acid 0·5 mg and choline 0·50 g.

† The ME was calculated from data provided by Feed Database in China.

### Measurement of growth performance

Feed intake and body weight were recorded on a per replicate basis on days 1, 21, 42 and 63. Mortality was checked daily, and dead birds were recorded and weighed to adjust estimates of gain, intake and feed conversion ratios, as appropriate. The average daily gain, average daily feed intake (ADFI) and feed:gain ratio (F:G) were calculated. At 63 d of age, birds were deprived of feed overnight and weighed immediately prior to slaughter.

### Sample collection

At 63 d of age, birds were deprived of feed overnight. Two male birds with similar body weights were chosen randomly from each pen. Birds were weighed, and blood samples were collected from the wing vein in a heparinised tube; serum was obtained after centrifugation (3000 ***g*** for 10 min), and the prepared serum was stored at –80°C to be used for anti-oxidative capacity tests, analysis of biochemistry parameters and ELISA. After blood collection, the birds were electrically stunned, exsanguinated and dissected by a trained team to collect tissue samples. The 0·5-cm upper-jejunum wall was fixed in 2·5 % glutaraldehyde (pH 7·4) and 4 % paraformaldehyde, respectively, and the mucosa of other jejunum segments and the middle segments of the duodenum were gently scraped. Then, the upper part of the caecum was tied with a string and snap frozen in N_2_. Moreover, the caecum contents were gently scraped with a blade and stored at –80°C until analysis.

One gram of duodenum and jejunal mucosa samples was homogenised with 9 ml of 0·9 % sterile normal saline on ice and centrifuged at 3500 ***g*** for 15 min at 4°C, respectively. The total protein concentration of the tissue supernatant was measured using a bicinchoninic acid (BCA) protein assay kit strictly according to the manufacturer’s protocols (Pierce). The prepared tissue supernatant was stored at –80°C and used in the anti-oxidative capacity test and ELISA.

### DNA extraction and 16S ribosomal RNA amplification sequencing

Total DNA was extracted and purified from approximately 200 mg of individual caecum contents using the QIAamp DNA Stool Mini kit (QIAGEN) according to the manufacturer’s instructions. Sequencing was performed at Novogene Bioinformatics Technology Co. Ltd. DNA was amplified using the 515f/806r primer set (515f: 5′-GTG CCA GCM GCC GCG GTA A-3′, 806r: 5′-XXX GGA CTA CHV GGG TWT CTA AT-3′). PCR was carried out in 30 μl reactions with 15 μl of Phusion^®^ High-Fidelity PCR Master Mix (New England Biolabs), 0·2 μm of forward and reverse primers and approximately 10 ng of template DNA. Thermal cycling consisted of initial denaturation at 98°C for 1 min, followed by thirty cycles of denaturation at 98°C for 10 s, annealing at 50°C for 30 s and elongation at 72°C for 30 s, with a final extension at 72°C for 5 min. PCR products were purified using the QIAquick Gel Extraction Kit (QIAGEN). Sequencing libraries were generated using the NEB Next^®^ Ultra™ DNA Library Prep Kit for Illumina (NEB) following the manufacturer’s recommendations, and index codes were added. The library quality was assessed on the Qubit@ 2.0 Fluorometer (Thermo Scientific) and Agilent Bioanalyzer 2100 system. Finally, the library was sequenced on an Illumina HiSeq platform and 250-bp paired-end reads were generated.

### Jejunum morphology

Jejunum samples were embedded in paraffin wax after dehydration and were sectioned at 5 μm on a rotary microtome. The sections were further stained with haematoxylin and eosin for morphological analysis, and digital images were obtained under a light microscope (Olympus)^(35)^. Transmission electron microscopy of the colonic tissue was conducted according to a previous study^(18)^.

### Detection of goblet cells

The morphology and distribution of goblet cells in the jejunum epithelium were observed by light microscopy after the sections were stained with periodic acid–Schiff’s reaction according to the previous procedures^(36)^. Moditec camera software was applied to take pictures. Five to ten complete intestine villi were selected from each tissue slice, and the number of goblet cells per 100 intestinal epithelial cells was counted.

### Terminal deoxynucleotidyl transferase-mediated deoxyuridine triphosphate-biotin nick end labelling assay

The extent of apoptosis in the jejunum tissue was estimated using the triphosphate-biotin nick end labelling method according to a previous study^(37)^. Briefly, sections were deparaffinised in xylene and hydrated in a graded alcohol series. Endogenous peroxidase was blocked with 2 % H_2_O_2_ in PBS. Terminal deoxynucleotidyl transferase enzyme and digoxigenin-labelled deoxyuridine triphosphate were applied to the sections for 1 h at 37°C. Sections were washed in wash buffer and treated with peroxidase-conjugated anti-digoxigenin antibody for 1 h at room temperature. Treatment with HistoMark Black chromogen solution was performed according to the manufacturer’s instructions. Sections were stained lightly in haematoxylin and eosin, dehydrated in an alcohol series, cleared in xylene and mounted in Permount.

### Relative protein expression by Western blot

The protein expression of mucin 2 (MUC-2) and tight junction proteins, including claudin-1, in the jejunum tissue was determined by Western blotting. Total protein extraction was performed using Tissue Protein Extraction Reagent (Thermo Pierce, 78510), and protein quantification was then performed using the BCA Quantitation Kit. After SDS-PAGE and membrane transfer, Tris-buffered saline (containing 5 % non-fat dry milk or bovine serum albumin) (Beyotime Biotechnology) was added to the membrane for blocking at room temperature for 1 h. The antibody (1:100) (Beyotime Biotechnology) was then added and incubated overnight at 4°C, followed by washing the membrane. Secondary antibody (goat anti-Mouse IgG (H + L)) (Beyotime Biotechnology) was added and incubated at room temperature for 1 h and then washed. SuperSignal^®^ West Dura Extended Duration Substrate was used for Western blot detection. The optical densities of the bands were analysed using Image J software. *β*-Actin was used as an internal control and was found to exhibit no differences between groups. The relative abundance of each target protein was expressed as the ratio of target protein:*β*-actin.

### Determination of serum and jejunum mucosal biochemistry parameters

Activities of lipase, trypsin and amylase in the duodenum mucosa were measured using colorimetric methods with a spectrophotometer (Biomate 5; Thermo Electron Corporation). The assays were conducted using assay kits according to the manufacturer’s instructions (Nanjing Jiancheng Bioengineering Institute). Individual serum samples were analysed for albumin (no. A028–1), globulin (no. H106), total protein (no. A045–2), uric acid (no. C012) and lysozyme (no. A050–1) using a kit package (Nanjing Jiancheng Bioengineering Institute). Absorbance was measured with an Infinite M200 Pro NanoQuant (Tecan).

### Anti-oxidative capacity measurements

The activities of catalase (no. A007–1), superoxide dismutase (SOD, no. A001–1) and glutathione peroxidase (GSH-Px, no. A005), the levels of total antioxidant capacity (T-AOC, no. A015–1) and malondialdehyde (no. A003–1) in serum and the jejunal mucosa were determined according to the manufacturer’s instructions (Jiancheng Bioengineering Institute).

### ELISA

The contents of secretory IL-6, TNF-*α*, IL-1*β*, IL-10, IgA and IgG in serum were determined colorimetrically using ELISA kits (Nanjing Jiancheng Institute of Bioengineering). IL-6, TNF-*α*, IL-1*β*, IL-10, secretory IgA (sIgA), endotoxin (ET) and d-lactic acid in the jejunum mucosal supernatant were also determined colorimetrically using ELISA kits (Nanjing Jiancheng Institute of Bioengineering) according to the manufacturer’s instructions. Briefly, serum and jejunum mucosal supernatant samples were pipetted into wells coated with antibodies specific for IL-6, TNF-*α*, IL-1*β*, IL-10, IgA, ET, d-lactic acid, IgG and sIgA. After incubation, biotinylated monoclonal secondary antibiotics were added, followed by streptavidin peroxidase. After incubation and rinsing, the bound cytokines were visualised by developing the peroxidase reaction through the addition of H_2_O_2_, and the absorbance of each well was determined with a SpectraMax M5^(38)^.

### Immunofluorescence

The distribution of the tight junction zonula occludens protein 1 and occludin in the jejunum tissue was observed by immunofluorescence. In brief, 5-μm tissue sections were incubated overnight at 4°C with rabbit anti-zonula occludens protein 1 antibody (Servicebio, GB11195) and rabbit anti-*β*-occludin antibody (Servicebio, GB11149). The sections were then incubated with Cyanine 3-conjugated goat anti-rabbit IgG (H + L) secondary antibody (Servicebio, GB21303). Finally, sections were stained with 4′,6-diamidino-2-phenylindole solution (Servicebio, G1012) for 10 min at room temperature in dark conditions, and the images were obtained using a Nikon Eclipse TI-SR fluorescence microscope together with a Nikon DS-U3 imaging system^(39)^.

### Statistical analysis

Raw reads were filtered using Cutadapt software (version 1.9.1), and the sequence database was built using the Ion Plus Fragment Library Kit, 48 rxns (Thermo Fisher Scientific). All clean reads were clustered into operational taxonomic units of 97 %, and representative sequences were annotated using an remote display protocol classifier (version 2.2). The top ten relative abundance values for all sample species at the phylum and genus levels were analysed using Mothur software. Subsequently, a ternary plot (R software, version 2.15.3) was used to analyse dominant microorganisms at the species level. Non-metric multi-dimensional scaling was used to assess the clustering of colonic microbial samples. *α*- and *β*-diversities were analysed based on the normalised data output and assessed using the Wilcoxon test. The UniFrac approach was used to estimate pairwise distances between samples and to establish *β*-diversity, which was visualised by the principal component analysis and clustering analysis. In addition, based on the taxonomic files obtained from Quantitative Insights In Microbial Ecology (QIIME) analysis, phylogenetic investigation of communities by reconstruction of unobserved states was performed online (http://huttenhower.sph.harvard.edu/galaxy/). The aforementioned process was similar to our previously reported process^(40)^.

All data were analysed using one-way ANOVA followed by Tukey’s multiple comparison using SPSS 23.0 software (SPSS Inc.). Data presented are shown as mean values and standard deviations, and values were considered significant at *P* < 0·05. Graphs were generated using GraphPad Prism 5.0 software.

## Results

### Growth performance of broilers

The effects of supplemental Ba, Enz or ER on the growth performance of Chinese yellow-feathered broilers are presented in Table 2. During the starter phase from 1 to 21 d, these parameters, including average daily gain, ADFI, F:G and mortality, were not different among the five groups. In the grower phase (days 22–42), all four treatments significantly decreased the F:G (*P* < 0·05) compared with that in the control group, similar to that with ER. During the final phase from 43 to 63 d, all four treatments significantly reduced mortality compared with that in the control group, and ER treatment significantly decreased the ADFI. Moreover, considering the entire growth period, the F:G and mortality of broilers subjected to the four treatments (Ba, Enz, Ba + Enz or ER) were significantly decreased when compared with those of the control group. These results indicated that supplementation with Ba, Enz and ER improved the growth performance of broilers.

**Table 2.** Effects of *Bacillus subtilis* DSM 29784, enzyme alone or in combination on the growth performance of broilers*

Items	A	B	C	D	E	sem	*P*
Starter phase (days 1–21)							
ADG (g/d)	19·08	19·35	18·37	18·78	18·35	0·451	0·142
ADFI (g/d)	30·94	31·21	29·57	29·98	29·20	0·729	0·045
F:G (g/g)	1·62	1·61	1·61	1·60	1·59	0·027	0·776
Mortality (%)	1·25	0·42	0·83	0·83	0·42	0·007	0·736
Grower phase (days 22–42)							
ADG (g/d)	35·25	37·06	35·91	36·16	35·30	1·509	0·750
ADFI (g/d)	90·67	88·51	86·21	86·28	85·45	3·002	0·415
F:G (g/g)	2·57^a^	2·39^b^	2·40^b^	2·39^b^	2·42^b^	0·059	0·023
Mortality (%)	3·38	2·51	2·52	1·68	2·51	0·009	0·430
Finisher phase (days 42–63)							
ADG (g/d)	47·49	46·84	45·93	45·61	43·04	2·249	0·358
ADFI (g/d)	140·27^a^	130·84^a,b^	128·95^a,b^	128·32^a,b^	119·99^b^	6·203	0·052
F:G (g/g)	2·95	2·80	2·82	2·81	2·82	0·140	0·812
Mortality (%)	4·38^a^	1·71^b^	1·74^b^	1·28^b^	0·84^b^	0·011	0·026
Whole phase (days 1–63)							
ADG (g/d)	33·94	34·42	33·40	33·51	32·23	1·041	0·326
ADFI (g/d)	86·48^a^	83·07^a,b^	81·00^a,b^	80·88^a,b^	77·66^b^	2·824	0·058
F:G (g/g)	2·55^a^	2·42^b^	2·43^b^	2·41^b^	2·41^b^	0·132	0·054
Mortality (%)	8·75^a^	4·58^b^	5·00^b^	3·75^b^	3·75^b^	0·015	0·015

ADG, average daily gain; ADFI, average daily feed intake; F:G, feed:gain ratio; A, control group; B, *Bacillus subtilis* DSM 29784 group; C, enzyme (6225 U/g xylanase and 3200 U/g *β*-glucanases) group; D, *Bacillus subtilis* DSM 29784 and enzyme group; E, enramycin group.

^a,b^ Mean values within the same row with unlike superscript letters were significantly different (*P* < 0·05).

* Each value represents the mean of six replicates (*n* 6). ADG = (last average weight – initial average weight)/feeding days; ADFI = total consumption/(total live chicken weight + total dead chicken weight – total initial weight); ADFI = ADG × ADFI.

### Immune system and intestinal barrier function

**Fig. 1.**
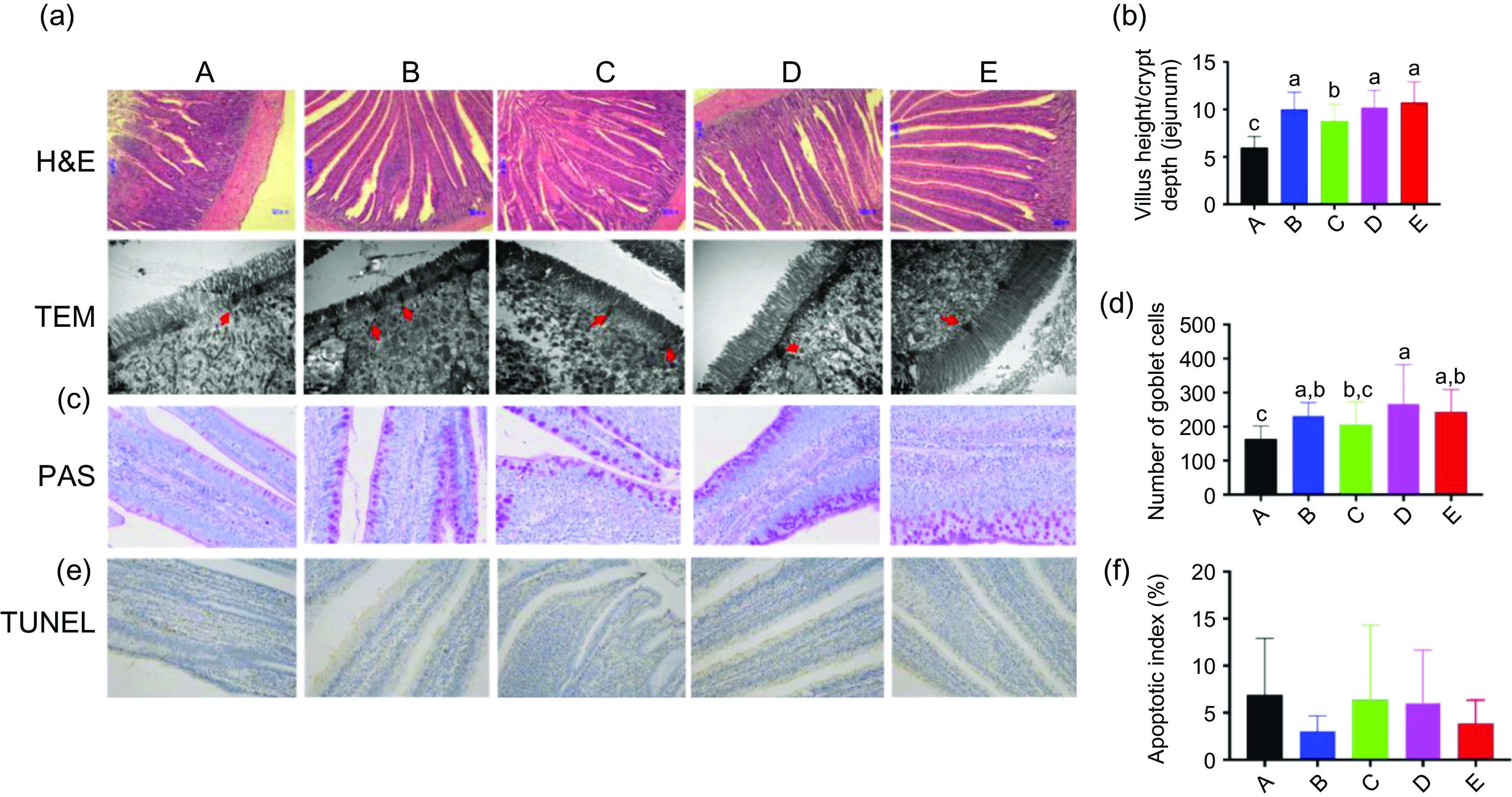
(a) Representative haematoxylin and eosin (H&E)-stained images (top, scale bars = 80 μm) and transmission electron microscopy images (bottom, scale bars = 1 μm; red arrows indicate epithelial cell junctions) of cross-sections of the bird’s jejunum. (b) Statistical analysis of the histological parameter ‘villus height:crypt depth ratio’ in the jejunum. (c) Representative images of periodic acid–Schiff’s (PAS) reaction-stained jejunum sections in broilers (400×). (d) Number of PAS-stained goblet cells. (e) Representative images of cross-sections of the broilers jejunum stained for triphosphate-biotin nick end labeling (TUNEL) (200×). (f) Quantification of TUNEL-(bottom) positive cells in the jejunum. A, control group (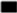); B, *Bacillus subtilis* DSM 29784 group (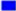); C, enzyme (6225 U/g xylanase and 3200 U/g *β*-glucanases) group (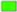); D, *Bacillus subtilis* DSM 29784 and enzyme group (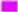); E, enramycin group (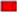). Values are means (*n* 12), with standard deviations represented by vertical bars. ^a,b,c^ Mean values with unlike letters between different groups were significantly different (*P* < 0·05).

**Fig. 2.**
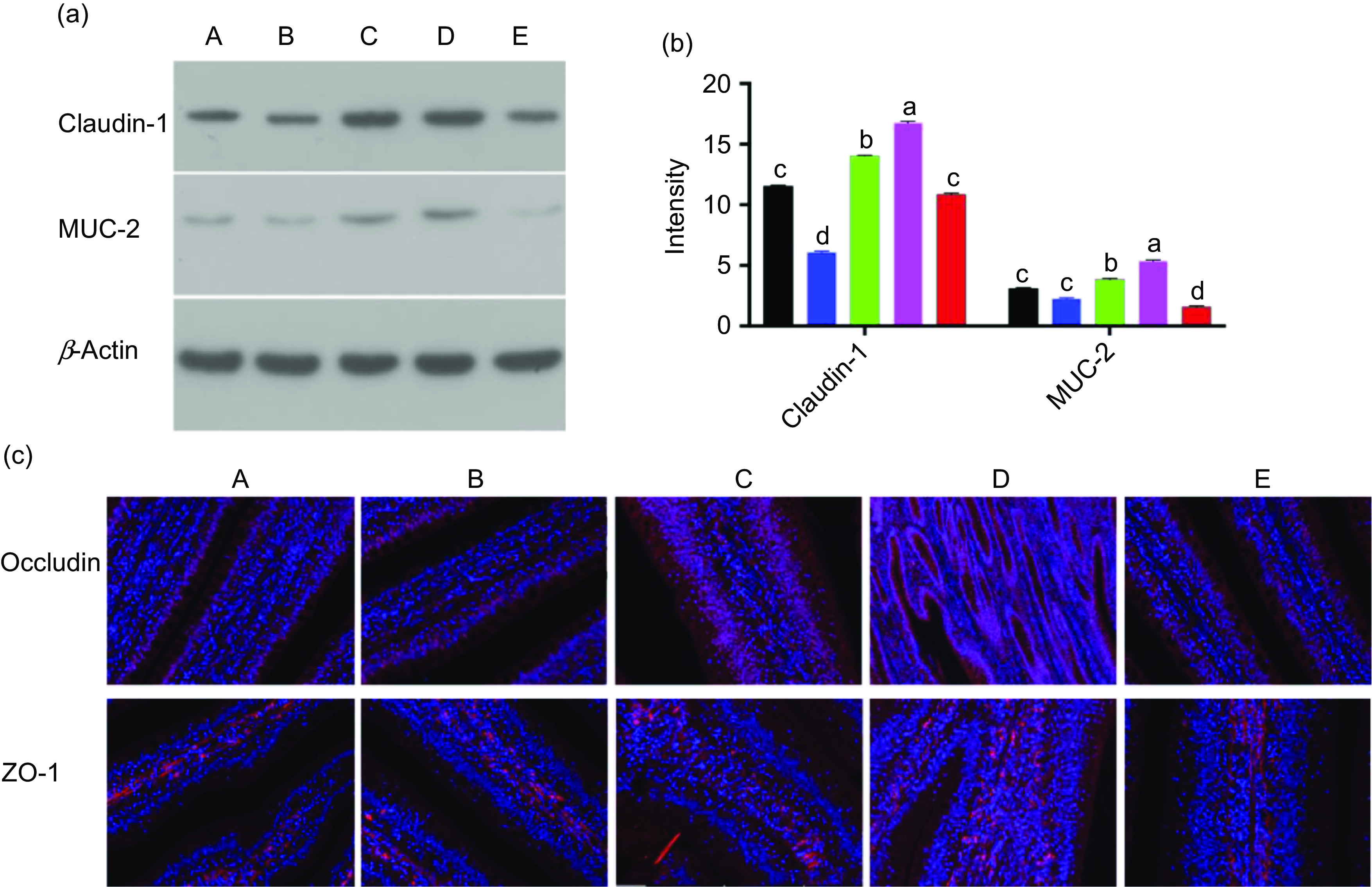
(a) Expression of mucin 2 (MUC-2) and claudin-1 in jejunum tissue was determined by Western blot. (b) Relative changes in the density of MUC-2 and claudin-1 were analysed. Data are presented as relative MUC-2 and claudin-1 band intensity normalised to *β*-actin band intensity. Values are means (*n* 12), with standard deviations represented by vertical bars. (c) The expression of occludin and zonula occludens protein 1 (ZO-1) was measured using immune staining. 4′,6-Diamidino-2-phenylindole was used for staining nucleus. A, control group (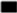); B, *Bacillus subtilis* DSM 29784 group (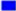); C, enzyme (6225 U/g xylanase and 3200 U/g *β*-glucanases) group (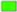); D, *Bacillus subtilis* DSM 29784 and enzyme group (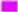); E, enramycin group (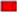). ^a,b,c,d^ Mean values with unlike letters between different groups were significantly different (*P* < 0·05).

Digital images and statistical analysis of haematoxylin and eosin-stained sections indicated that the intestinal structure of animals of all treatment groups presented with normal intestinal morphology and a significantly increased villus height:crypt depth ratio compared with that in the control group. Furthermore, all treatment groups showed increased heights and quantity of microvilli, as well as the distribution of epithelial cell junctions in the jejunum (Fig. 1(a) and (b)). Moreover, digital images and statistical analysis of periodic acid–Schiff-stained sections showed that Ba and BE, similar to that with ER, increased the numbers of goblet cells (Fig. 1(c) and (d)). The nuclei of triphosphate-biotin nick end labelling-positive cells were stained brown. Triphosphate-biotin nick end labelling-positive cells were mainly distributed in the apical region of villi (Fig. 1(e)). Compared with that in control broilers, the number of triphosphate-biotin nick end labelling-positive cells in the other four treatment groups showed no significant changes (Fig. 1(f)). To further investigate the effect of Ba, Enz or ER on the intestinal physical barrier function, the relative protein expression of MUC-2 and the tight junction protein-related protein claudin-1 were examined. Enz and BE significantly increased MUC-2 and claudin-1 expression in the jejunal mucosa compared with that in the control or ER group (*P* < 0·01), whereas feed supplemented with Ba did not induce any change in MUC-2 expression and reduced claudin-1 expression compared with that in the control group (Fig. 2(a) and (b)). To confirm the impact of Ba, Enz or ER on the expression of tight junction and adherence junction proteins, we further performed immunofluorescence analysis and the resulting images demonstrated a similar result to epithelial cell protein junction expression in the jejunal mucosa (Fig. 2(c)).

### Biochemical indices in the duodenum and serum

**Fig. 3.**
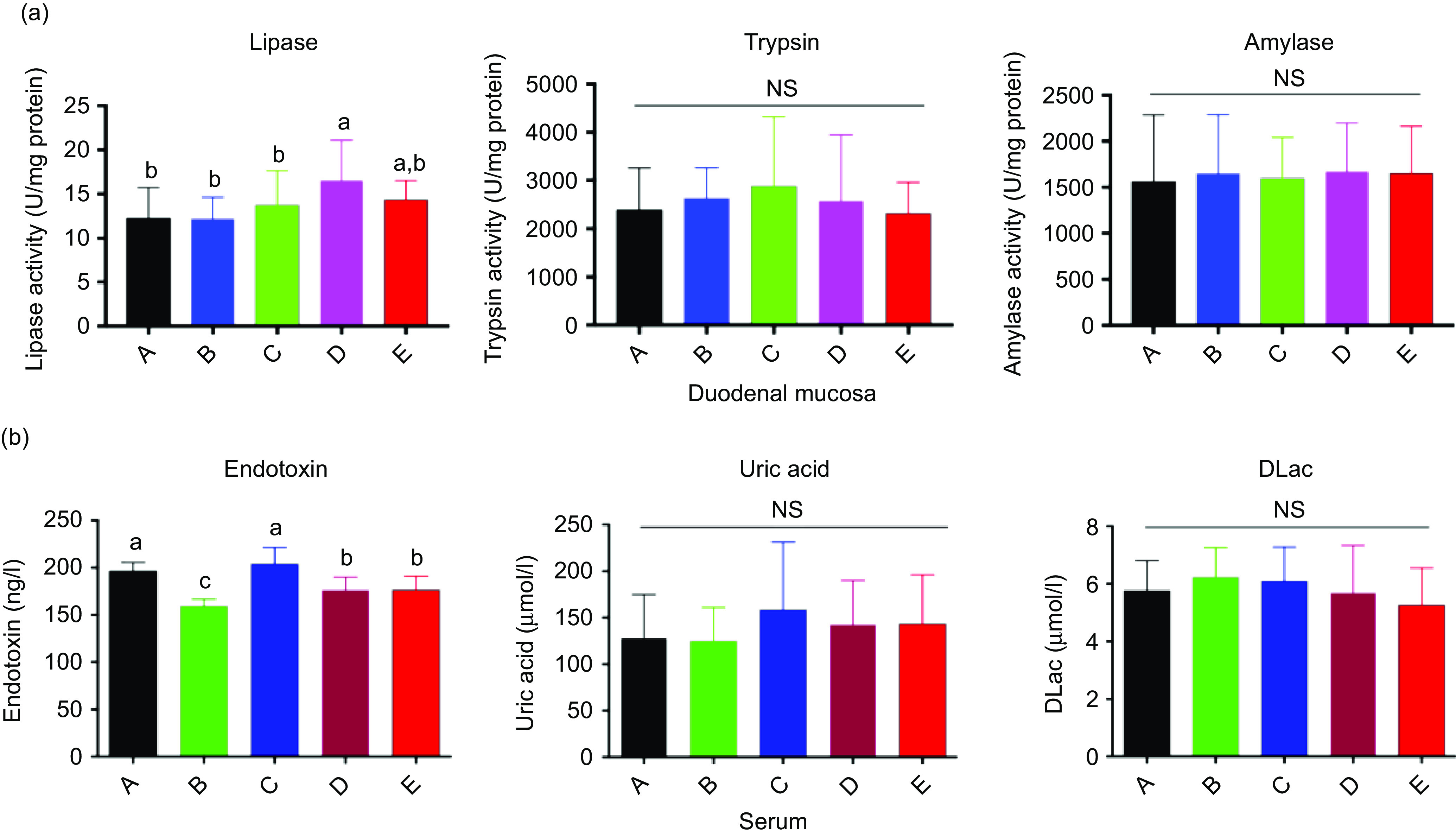
Duodenal mucosa (a) and serum (b) biochemistry parameters of broilers chickens supplemented with *Bacillus subtilis* DSM29784, enzyme alone or in combination. A, control group (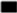); B, *Bacillus subtilis* DSM 29784 group (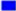); C, enzyme (6225 U/g xylanase and 3200 U/g *β*-glucanases) group (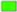); D, *Bacillus subtilis* DSM 29784 and enzyme group (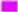); E, enramycin group (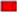). Values are means (*n* 12), with standard deviations represented by vertical bars. ^a,b,c^ Mean values with unlike letters between different groups were significantly different (*P* < 0·05).

Compared with that in the control group, BE significantly increased lipase activity in the duodenum among the five treatments (Fig. 3(a); *P* < 0·05), whereas no differences were observed in the activities of trypsin and amylase. The serum ET levels in both the Ba and BE groups were markedly decreased by 18·92 and 10·14 %, respectively. However, no significant changes were observed in the contents of uric acid and d-lactic acid in the serum of broilers among the five treatment groups (Fig. 3(b)).

### Antioxidant-related parameters in serum and jejunum mucosa

**Fig. 4.**
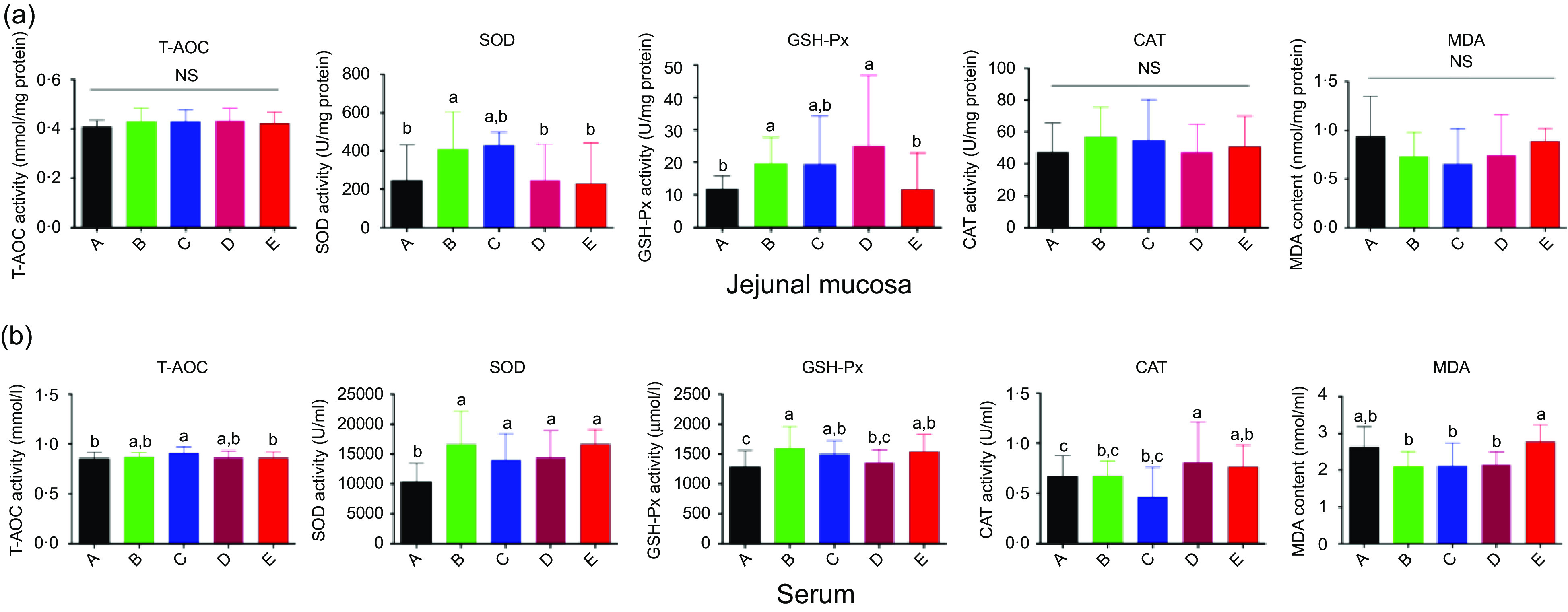
Effects of *Bacillus subtilis* DSM29784, enzyme alone or in combination on anti-oxidative capacity of broilers. The activities of anti-oxidative enzymes and levels of total antioxidant capacity (T-AOC) and malondialdehyde (MDA) were determined in jejunal mucosa (a) and serum (b), respectively. SOD, superoxide dismutase; CAT, catalase; GSH-Px, glutathione peroxidase. A, control group (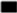); B, *Bacillus subtilis* DSM 29784 group (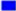); C, enzyme (6225 U/g xylanase and 3200 U/g *β*-glucanases) group (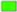); D, *Bacillus subtilis* DSM 29784 and enzyme group (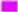); E, enramycin group (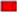). Values are means (*n* 12), with standard deviations represented by vertical bars. ^a,b,c^ Mean values with unlike letters between different groups were significantly different (*P* < 0·05).

Antioxidant parameters in the jejunum are shown in Fig. 4(a). Ba significantly increased SOD activity (*P* < 0·05). Further, the GSH-Px activity in both the Ba and BE groups was higher than that in the control or ER group (*P* < 0·05). However, no significant difference was found among the Ba, Enz, BE and ER groups in other antioxidant parameters including T-AOC, catalase and MAD (Fig. 4(a)).

Fig. 4(b) represents the anti-oxidative parameters of serum. Compared with that in the control group, Enz significantly increased serum T-AOC (*P* < 0·05), and BE increased catalase activity (*P* < 0·05), whereas the GSH-Px activity in the Ba and Enz groups was significantly increased (*P* < 0·05). In addition, the serum SOD activity was significantly increased, whereas the malondialdehyde level was significantly decreased in the Ba, Enz and BE treatment groups (*P* < 0·05; Fig. 4(b)).

### Immune response in serum and jejunum mucosa

**Fig. 5.**
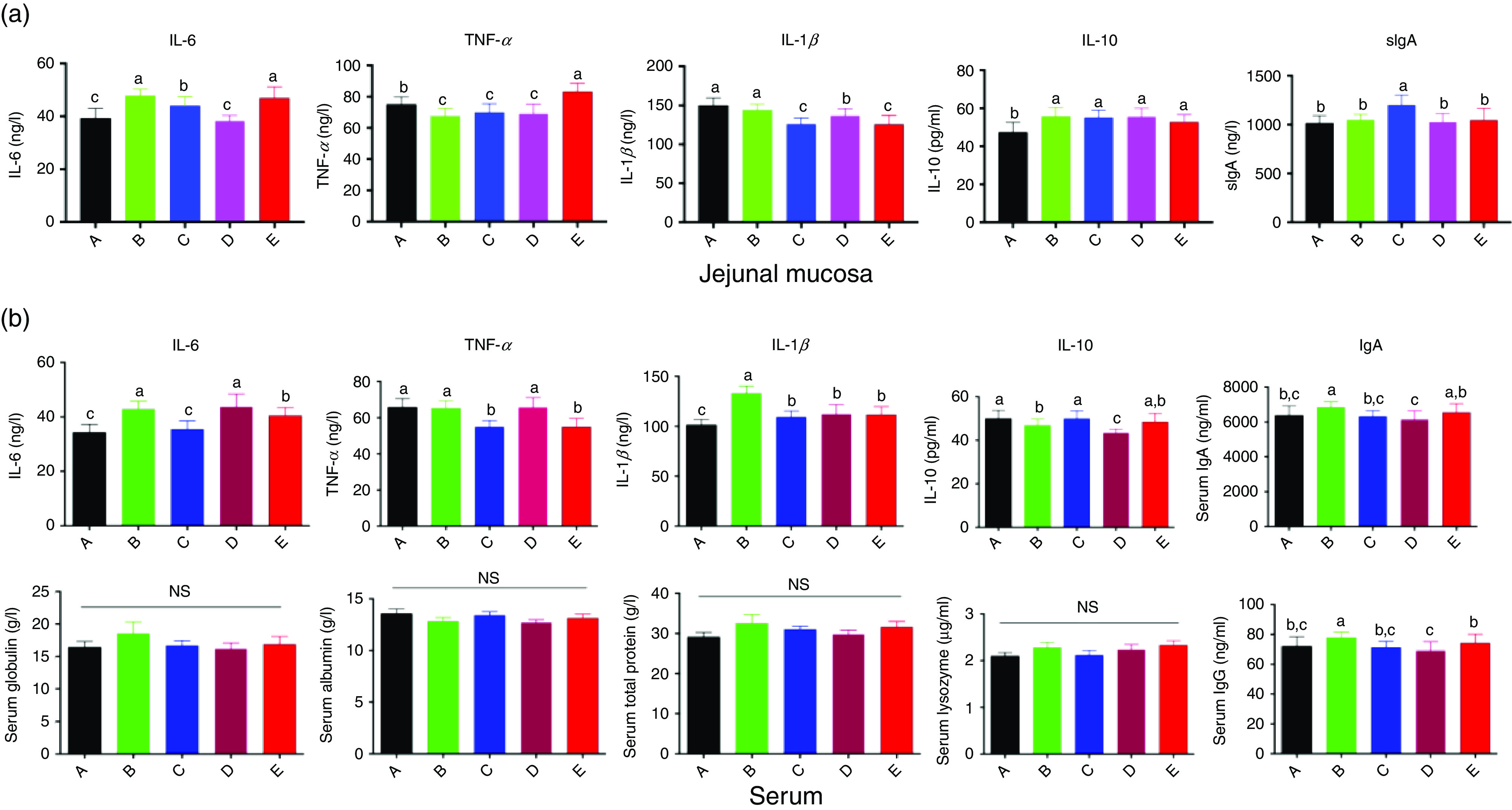
Effects of *Bacillus subtilis* DSM29784, enzyme alone or in combination on immune response of broilers in jejunal mucosa (a) and serum (b), respectively. A, control group (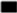); B, *Bacillus subtilis* DSM 29784 group (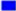); C, enzyme (6225 U/g xylanase and 3200 U/g *β*-glucanases) group (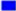); D, *Bacillus subtilis* DSM 29784 and enzyme group (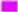); E, enramycin group; sIgA, secretory IgA. Values are means (*n* 12), with standard deviations represented by vertical bars. ^a,b,c^ Mean values with unlike letters between different groups were significantly different (*P* < 0·05).

Cytokine secretion in the jejunum mucosa is shown in Fig. 5(a). Compared with that in the control or ER group, the IL-6 level in both the Ba and Enz groups was markedly increased by 22·22 and 12·50 %, respectively, whereas IL-1*β* was decreased in the Enz and BE groups by 16·07 and 8·62 %, respectively. TNF-*α* was significantly decreased (*P* < 0·05), whereas IL-10 was increased (*P* < 0·05) in the Ba, Enz and BE treatment groups (Fig. 5(a)). sIgA in the Ba group was higher than that in the other four groups (*P* < 0·05; Fig. 5(a)).

The immune parameters of serum are presented in Fig. 5(b). Compared with that in the control group, the serum IL-6 level in both the Ba and BE groups was significantly increased by 25·18 and 23·92 %, respectively, and IL-10 was decreased by 5·90 and 13·01 %, respectively. Serum IL-1*β* in the Ba group was higher than that in the other four groups, whereas TNF-*α* levels were significantly decreased compared with those in the control group. In addition, IgA and IgM in the Ba group were higher than that in the other four groups. No significant changes were observed in serum globulin, albumin and total protein levels and in the activity of lysozyme among the five treatment groups.

### Microbial community structure of the caeca

**Fig. 6.**
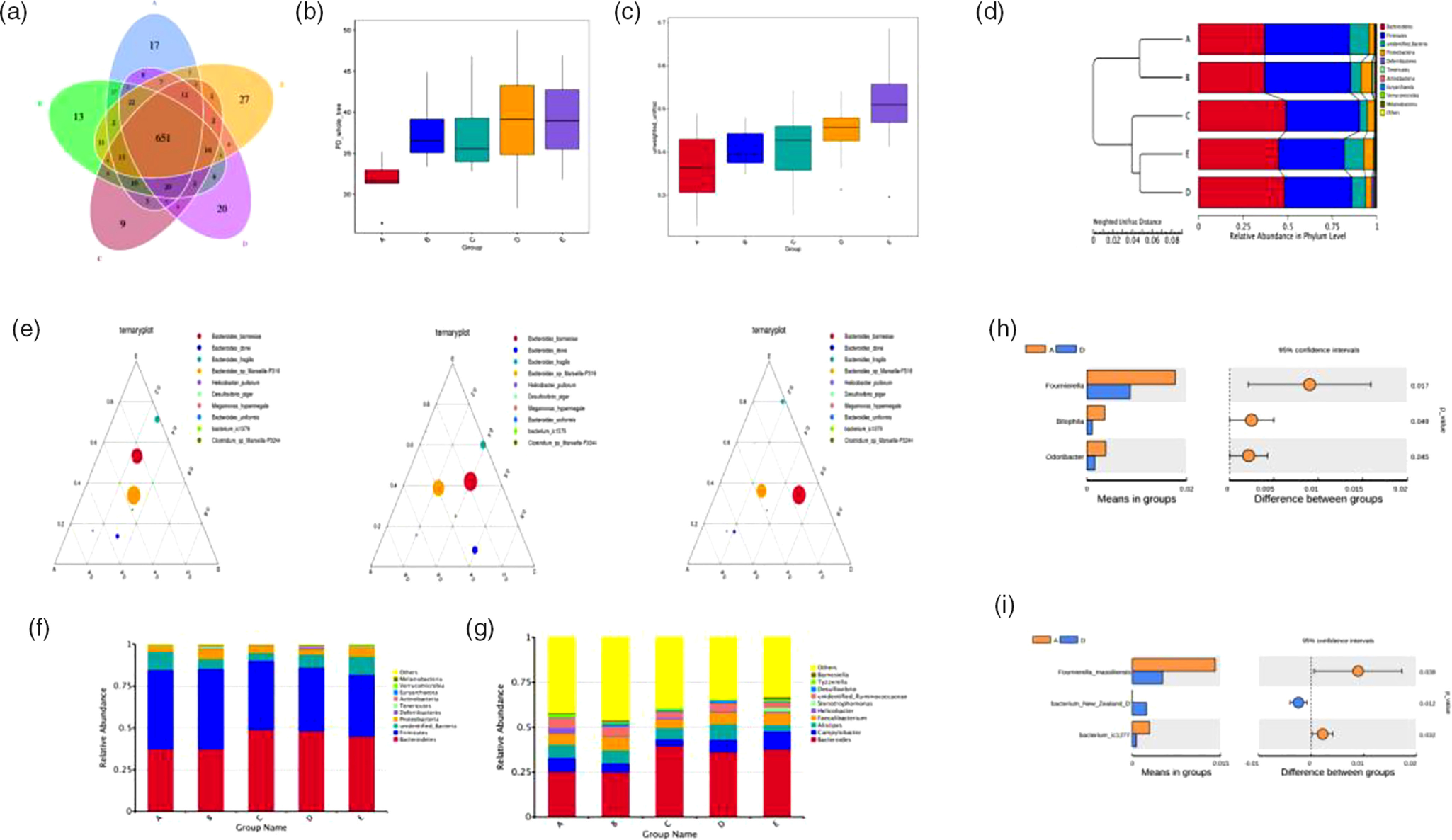
The caecal bacterial community of broilers fed dietary *Bacillus subtilis* DSM 29784, multi-enzyme or enramycin supplementation among the five treatments (A, B, C, D and E). (a) The Venn diagram presents overlaps among the A–E treatments, as well as the unique operational taxonomic units for each group. (b) *α*-Diversity of gut microbial was analysed among A–E treatments by determination of principal dimension (PD) whole tree index. (c) *β*-Diversity based on unweighted UniFrac of caecum microbiota among the five groups. (d) Unweighted pair group method with arithmatic mean (UPGMA) clustering tree with weighted UniFrac distances. (e) Ternary plot of A–B–E, A–C–E and A–D–E. Different size of circles corresponds to the abundance of bacteria. The relative abundances of species at the phylum level (f) and genus level (g), respectively. The *t* test analyses of the abundances between A and D in differential species: (h) genus level and (i) species level. A, control group; B, *Bacillus subtilis* DSM 29784 group; C, enzyme (6225 U/g xylanase and 3200 U/g *β*-glucanases) group; D, *Bacillus subtilis* DSM 29784 and enzyme group; E, enramycin group.

The number of operational taxonomic units in the control group was 801, whereas that in the other four experimental groups was 799, 764, 786 and 794, respectively. Operational taxonomic units in common were 651 and the common ratio was 81·27 %, suggesting that there were still a few differences (Fig. 6(a)). Furthermore, we determined the bacterial richness and diversity of the caeca microbiota in broilers among the five treatment groups. The principal dimension (PD) whole tree index was higher in the BE and ER treatment groups than in the control group (Fig. 6(b)). Moreover, BE-treated broilers exhibited a significantly higher *β*-diversity parameter based on unweighted UniFrac than the control or Ba treatment groups (Fig. 6(c)). Clustering analysis revealed short UniFrac distances among the Enz, BE and ER treatment groups and long branches separating the samples from the control and those three groups (Fig. 6(d)). However, there was no significant difference between the control and Ba treatment groups. The dissimilarity in the caeca microbial community was greatest between the control and BE treatment groups, as with ER. Ternary plot analysis indicated that *Bacteroides barnesiae*, *Bacteroides dorei*, *Bacteroides fragilis* and *Bacteroides* sp. dominated the caecal microbial community of all broilers on day 63. Notably, the antibiotic-treated group was more similar to the Ba or Enz treatment groups than to the control group (Fig. 6(e)).

The dominant phyla in the caeca across five treatment groups were Bacteroidetes, Firmicutes and Proteobacteria, together accounting for more than 90 % of the total sequences (Fig. 6(f)). Compared with those in the control group, birds in the Enz and BE treatment groups at these three levels had higher relative abundances of Bacteroidetes and Proteobacteria and a lower relative abundance of Firmicutes in the caeca microbiota, as with ER. However, the greatest abundance of Proteobacteria was observed in the Ba treatment group, whereas no significant changes were observed between the control and Ba treatment groups in the relative abundance of Bacteroidetes and Firmicutes (Fig. 6(f)). At the genus level, an increase in the abundance of *Bacteroides* was also detected in the other four treatment groups compared with that in the control group (Fig. 6(g)). Further analysis revealed that BE treatment resulted in lower abundances of *Fournierella*, *Bilophila*, *Odoribacter*, *Fournierella massiliensis*, *bacterium New Zealand D* and *bacterium ic1277* in the caecum compared with those in the control group (Fig. 6(h) and (i)).

**Fig. 7.**
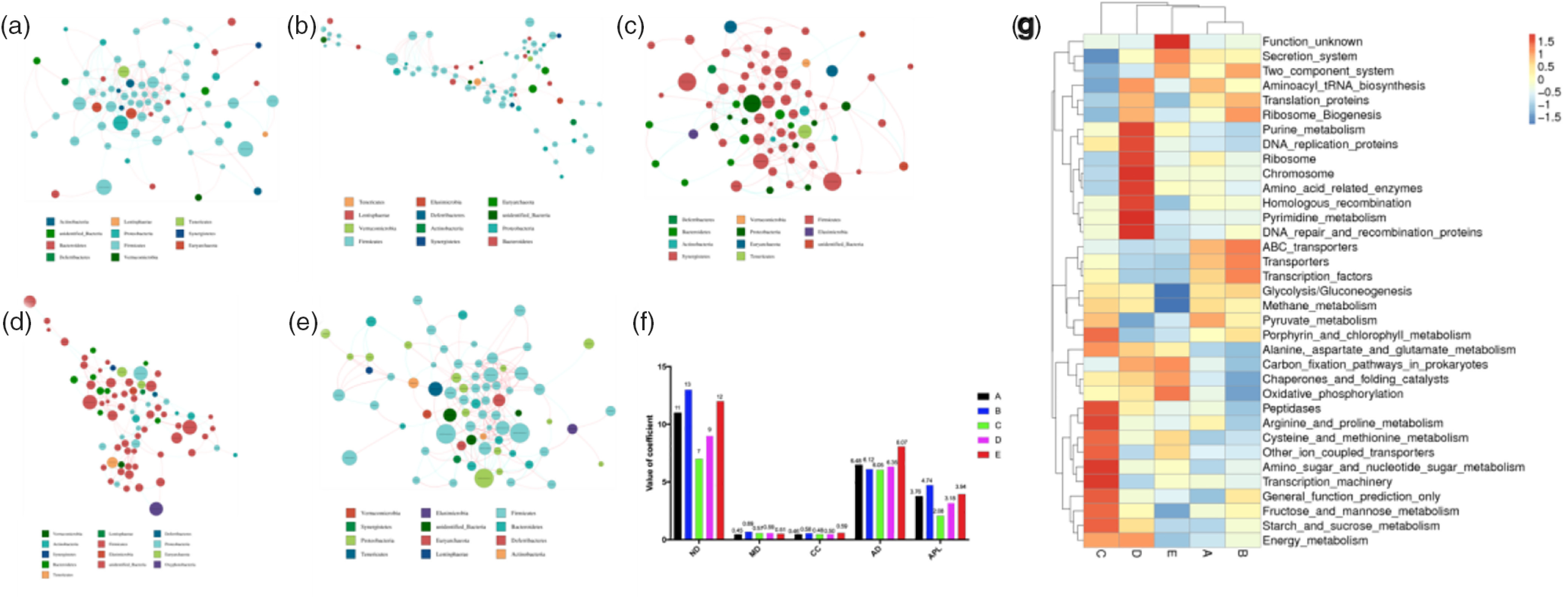
(a–e**)** Network analysis of microbial community in A, B, C, D and E groups based on the calculation of Spearman’s correlation coefficients. Nodes indicate taxonomic units at the genus level, while red lines indicate positive correlations and blue lines indicate negative correlations. The colour of a node represents the phylum to which it belongs, and the size of a node represents the relative abundance of a genus. The thickness of a line indicates the strength of correlation. (f) Typical coefficients derived from network analysis in the five groups (CC, clustering coefficient, GD, graph density, AD, average degree, APL, average path length). (g) Functional abundance cluster analysis. A, Control group; B, *Bacillus subtilis* DSM 29784 group; C, enzyme (6225 U/g xylanase and 3200 U/g *β*-glucanases) group; D, *Bacillus subtilis* DSM 29784 and enzyme group; E, enramycin group.

To further determine the relationships among different microbes in the five treatment groups, we also performed network analysis of gut microbiota by calculating Spearman’s correlation coefficients among all genera. Our results revealed a larger proportion of negative correlations in the microbial community and fewer interactions of gut microbiota in the Enz, BE or ER treatment groups as indicated by lower coefficients of network analysis including cluster coefficients network diameter, modularity, clustering coefficient average degree and average path length/mean distance. In contrast, higher coefficients of network analysis were observed in the Ba treatment group based on higher network diameter, modularity, clustering coefficient and average path length (Fig. 7(a)–(f)).

### Predicted function of caecal microbiota

To investigate the differences in microbiota functions among the five groups, we performed a functional analysis of microbes (using phylogenetic investigation of communities by reconstruction of unobserved states). Third-level Kyoto Encyclopedia of Genes and Genomes analysis showed that purine metabolism, homologous recombination, chromosome, energy metabolism, amino acid-related enzymes, ribosome biogenesis, pyrimidine metabolism and DNA replication, repair and recombination proteins were enriched in BE-treated broilers compared with that in the control or ER-treated birds. Meanwhile, amino sugar and nucleotide sugar metabolism, cysteine and methionine metabolism, transcription machinery, starch and sucrose metabolism, peptidases, general function prediction only, fructose and mannose metabolism, and arginine and proline metabolism pathways were enriched in the Enz-treated birds compared with those in the control or ER-treated broilers. Moreover, the microbiota of the Ba treatment group displayed a higher abundance of functions involving metabolic pathways such as transporters, transcription factors and ribosome biogenesis, compared with those in the other four groups (Fig. 7(g)).

## Discussion

As food safety and environmental contamination are receiving increasing attention, antibiotic replacement has become a trend. Probiotics and feed enzymes have been widely used as antibiotic replacements to enhance animal growth and intestinal health^(41,42)^. Çınar *et al*.^(43)^ demonstrated significant improvement in the feed conversion ratio when prebiotics and probiotics were supplemented together, suggesting synergism between them. Moreover, a glucose oxidase and *Bacillus amyloliquefaciens SC06* combination had a better anti-apoptotic effect than glucose oxidase alone^(18)^. A combination of the probiotic strains *Lactobacillus plantarum* KY1032 and *Lactobacillus curvatus* HY7601 was more effective in inhibiting the gene expression of various fatty acid synthesis enzymes in the liver, concomitant with decreases in fatty acid oxidation-related enzyme activities and their gene expression^(44)^.

During the starter phase from 1 to 21 d, there was no significant effect on average daily gain, ADFI, F:G and mortality due to Ba or Enz treatment. In contrast, the current study showed that all treatments significantly decreased the F:G during the grower phase compared with that in the control group, similar to that with ER. Moreover, considering the whole growth period, the F:G and mortality of broilers in all treatment groups decreased. These results might be due to the fact that Chinese yellow-feathered broilers mature at a moderate pace and grow slowly in the start phase. In addition, barley was used in the diet during the growth and final phases, and the improvement of the growth performance of broilers might be due to the addition of Ba or Enz. As a green feed additive, *B. subtilis* has been widely promoted as an alternative to replace in-feed antibiotics due to its abilities to improve livestock production and efficiency^(45)^. In addition, many studies have shown that xylanase and *β*-glucanase can stimulate the growth response and feed efficiency of chickens^(28)^, turkeys^(30)^, pigs^(46)^ and fish^(25)^. Our data are in agreement with these findings. However, ER treatment significantly decreased the ADFI compared with that in the control group during the final phase; this might be due to the significantly increased mortality in the control group.

The concept of ‘gut health’ is complex but is now well recognised as a key driver of animal performance^(47)^. The intestinal barrier comprises four main components, the physical, chemical, immunological and microbiological barriers^(48)^. The health of the gastrointestinal tract affects the digestion, absorption and metabolism of nutrients, disease resistance and immune responses^(49,50)^. A complete small intestinal structure is important for digestive and absorptive functions of the small intestine and is closely related to the morphological changes in small intestinal villus length and crypt depth^(51)^. Moreover, the intestinal epithelium serves as a protective barrier and plays a beneficial role in nutrient absorption; its morphology is also used to assess intestinal development and function^(40)^. Al-Fataftah & Abdelqader^(52)^ indicated that dietary supplementation with *B. subtilis* enhances the intestinal barrier function in animal models. In the present study, the intestinal morphology indicated that all treatments had positive effects on the tight junctions or microvillus length, width and density compared with those in the control group. Goblet cells synthesise and secrete lubricant mucus that forms a mucus layer in the small intestine to protect the epithelial cells^(53)^. In the present study, we observed that the number of goblet cells was obviously increased in the jejunum of chickens fed the Ba, BE or ER diets when compared with that in chickens fed the control diet (Fig. 1(d)). In addition, tight junctions are essential components of intestinal mucosal barriers, providing paracellular permeability^(54)^. The MUC-2 protein is a major component of the intestinal mucus layer, which plays an important role in preventing intestinal damage by bacteria and lubricating the small intestine to maintain mucosal barrier functions^(55)^, whereas a decrease in MUC-2 induces an inflammatory immune response^(56)^. In the present study, our results showed that the relative protein expression of MUC-2 and tight junction protein-related protein claudin-1 was increased in the Enz and BE groups compared with that in the control or ER group. This finding demonstrated that Enz or BE might enhance the intestinal barrier function.

Endotoxins are also referred to as lipopolysaccharides, which are cell wall components of Gram-negative bacteria. Minor or trace amounts of ET, even at the pico-gram scale, have the potential to elicit an inflammatory response in the host once they enter the interior circulation^(57,58)^. However, ET concentrations can become fatal if they reach up to 1 g, especially in the intestines^(59)^. A previous study reported that damage to the intestinal tract epithelium leads to the enormous absorption of ET from the intestinal tract^(57)^. In the present study, reduced serum ET content was found in the Ba, BE and ER groups, which indicated that Ba, BE and ER might improve the integrity of the intestinal tract epithelium.

The intestinal environment plays a critical role in maintaining good health^(60)^. As is known, an increase in lipase activity leads to more effective fat absorption^(61)^. Yango *et al.* demonstrated that lyophilised photosynthetic bacterial cells at 1 g/kg and lyophilised *Bacillus* sp. at 1 g/kg in combination highly increase the amylase and lipase enzyme activities of common *Cyprinus carpio* ^(62)^. However, Ogawa *et al*.^(63)^ indicated that *Lactobacillus gasseri* SBT2055 significantly decreases lipase activity to increase the size of fat emulsion droplets and suppress lipid absorption. In the present study, we found that broilers in the BE group had much higher activity of lipase in the contents of the jejunum than broilers fed a control diet, whereas no difference was found with Ba or Enz supplementation alone.

Various stressful conditions enhance the production of free radicals and restrict the growth performance of chickens in the poultry industry^(64)^. The excessive formation of free radicals contributes to a detrimental biological condition, namely oxidative stress, which gives rise to performance losses, meat deterioration, intestinal dysfunction and immunosuppression of broilers^(65–67)^. Antioxidants interact in a complex network to recycle and regenerate one another and protect against oxidative stress-induced intestinal diseases^(68)^. Probiotics are regarded as a natural source that can scavenge excess free radicals, as well as improve the antioxidant capacity of animals^(69)^. The antioxidant system includes both natural and synthetic anti-oxidative enzymes^(70)^. Cellular resistance to oxidative agents is controlled by several key enzymes such as T-AOC, GSH-Px, SOD and catalase^(71)^. In addition, malondialdehyde is associated with lipid peroxidation and avian stress^(72)^. In the present study, Ba administration increased the activities of GSH-Px and SOD in the serum and the jejunum (Fig. 4(a) and (b)), whereas Enz increased the activities of serum T-AOC, SOD and GSH-Px. However, BE did not have a better effect than Ba or Enz alone on antioxidant capacity. These results indicate that Ba and Enz, alone or in combination, enhance the antioxidant capacity of broilers.

Intestinal dysbiosis associated with immunological deregulation, leaky gut, bacterial translocation and systemic inflammation has been associated with autoimmune diseases^(73)^. The innate immune system is composed of a network of cells including neutrophils, natural killer and natural killer T cells, monocytes/macrophages and dendritic cells that mediate the earliest interactions with pathogens^(74)^. Poultry can generate cytokines inside their bodies, which can influence the immune system. Cytokines are of importance within the innate immune system^(75)^. As shown in Fig. 5, supplementation with Ba or Enz significantly increased the anti-inflammatory cytokine IL-10 in the jejunum and reduced the pro-inflammatory cytokine TNF-*α*, with increased IL-6 content; moreover, Enz decreased IL-1*β* secretion and resulted in the highest sIgA level among the five groups. In addition, Ba significantly increased serum IgA and IgG contents compared with those in the control or ER group. Inflammatory cytokine production can enhance the immune response to protect against pathogen invasion, whereas an excessive inflammation response leads to tissue damage^(76)^. In addition, sIgA can protect the intestinal tract from dietary and microbial antigens^(77)^. sIgA inhibits the adherence and invasion of potentially harmful antigens into the mucosa and neutralises toxins and virulence factors from microbial pathogens^(78)^. Taken together, probiotics or enzymes might activate the immune response to protect against pathogen invasion and hence maintain intestinal homeostasis by a balance between pro-inflammatory and anti-inflammatory responses.

Gut microbiota might function to prevent pathogens from colonisation of the intestinal tract. The importance of commensal gut microbiota is highly important for the normal functioning of the immune apparatus of the gut^(31,79)^. Various studies have demonstrated that probiotics can positively regulate the composition of intestinal microbiota^(12,80)^. Lei *et al*.^(81)^ demonstrated that *B. amyloliquefaciens* can dramatically decrease the population of *Escherichia coli* and increase *Lactobacillus* populations in the caecum. Dietary supplementation with probiotics increases the *Bifidobacterium* and *Lactobacillus* concentrations^(82)^ and increases the activities of caecal microflora^(83)^. The administration of *Clostridium butyricum* was found to decrease *E. coli* and *Salmonella* and *Clostridium perfringens* counts and increase *Lactobacillus* and *Bifidobacterium* counts in the caeca^(84)^. In addition, xylanase and *β*-glucanase supplementation influences the microbiota of broiler chicken caeca and might reduce potentially pathogenic Enterobacteriaceae populations^(29)^. Our work demonstrated that supplementation with Ba, ER or BE alters the structure of caecal microflora, with observably distinctive operational taxonomic units appearing and with markedly different community structures based on the principal component analyses. The *α*-diversity according to the PD whole tree and *β*-diversity parameter based on unweighted UniFrac were significantly increased in the BE group; moreover, the clustering analysis revealed short UniFrac distances between the BE and ER groups. Kristensen *et al*.^(80)^ demonstrated that probiotic supplementation significantly modifies the overall structure of the faecal bacterial community in terms of *β*-diversity when compared to that with the placebo. According to our findings, the Ba and Enz combination had a better effect on intestinal microbiota richness. The results showed that the relative abundances of Bacteroidetes and Proteobacteria in the caeca of broilers were increased, as with ER, and Bacteroidetes and Firmicutes were the two most abundant phyla of these broiler chickens. This result was similar to that of a previous study indicating that Bacteroidetes and Firmicutes are associated with an absolute advantage in piglet guts^(85)^. Firmicutes can maintain intestinal health by producing SCFA, inhibiting inflammation, providing energy for intestinal epithelial cells and contributing to animal energy metabolism^(86,87)^. However, Proteobacteria are a potential diagnostic signature of dysbiosis and risk of disease, as a sustained increase in the abundance of this phylum often can result in an imbalanced gut microbiota^(88)^. Intestinal microbiota are known to have physiological effects on a host in terms of metabolising dietary nutrients, producing SCFA from indigestible carbohydrates, synthesising amino acids and vitamins and regulating metabolism^(87,89)^. According to our findings, purine metabolism, homologous recombination, chromosome, energy metabolism, amino acid-related enzymes, ribosome biogenesis, pyrimidine metabolism and DNA replication, repair and recombination proteins were enriched in BE-treated broilers.

In summary, dietary treatment with Ba, Enz or BE was found to have beneficial effects on the growth performance of broilers. This enhancement was associated with the positive influence of tract digestibility and anti-oxidative capacity, immune function and intestinal microbiota. In addition, the results suggested that BE exerts a better effect, compared to that with Ba or Enz alone, on the growth performance, digestive enzyme activity and the population of intestinal microbiota. Therefore, according to our research, Ba or Enz could be used as an alternative to antibiotics in broiler chickens.
